# Insulin-like growth factor-1 coordinately induces the expression of fatty acid and cholesterol biosynthetic genes in murine C2C12 myoblasts

**DOI:** 10.1186/1471-2164-9-535

**Published:** 2008-11-11

**Authors:** C Ramana Bhasker, Theodore Friedmann

**Affiliations:** 1 Center for Molecular Genetics, Department of Pediatrics, School of Medicine, University of California, San Diego, La Jolla CA 92093-0634 USA

## Abstract

**Background:**

We present evidence that a major aspect of the mechanism of acute signal transduction regulation by insulin-like growth factor-1 (IGF-1) in cultured murine myoblasts is associated with a broad perturbation of many components of cholesterol and fatty acid biosynthetic pathways.

**Results:**

We have used microarray transcriptional analysis to examine the acute effects of IGF-1 on global patterns of gene expression in C2C12 myoblasts and have identified approximately 157 genes that are up-regulated and 75 genes down-regulated from 2- to 6-fold after treatment with IGF-1. Of the up-regulated genes, 19 genes are associated with cholesterol biosynthesis and 5 genes specify aspects of fatty acid biosynthesis. In addition 10 recognized transcription factors are significantly induced by IGF-1 at 1 hour.

**Conclusion:**

The SREBPs, important regulators of fatty acid and cholesterol biosynthesis, operate via a post-transcriptional route and no significant transcriptional induction was observed in the 4 hr of IGF-1 treatment. Since there are no prior reports of significant and coordinated perturbations of fatty acid and cholesterol biosynthetic pathways with IGF-1 in muscle cells, these findings provide a substantive expansion of our understanding of IGF-1 action and the signal transduction pathways mediated by it, its variants and insulin.

## Background

IGF-1 is a multifunctional polypeptide hormone that plays a central role in controlling somatic growth and participates in muscle development, maintenance and regeneration [1-3]. Several forms of IGF-1 exist as splicing variants that are differentially distributed in different cell types and may have associated cell type specific functions. Whilst the main source of IGF-1 synthesis is the liver, its target cells are mostly in the liver and muscle. Since muscle constitutes over 40 percent of the body mass, it becomes an important tissue to investigate the effects of IGF-1. IGF-1 influences the development and maintenance of muscle cells at least partly through the early activation of signal transduction pathway proteins leading to the induction of specific transcription factors that consequently trigger downstream target genes. Disruption of IGF signaling by targeted knockout of the IGF-IR gene causes growth impairment and severe skeletal muscle hypoplasia [4]. Conversely, over-expression of IGF-1 in skeletal muscle stimulates hypertrophy and also counteracts loss of muscle mass that occurs during aging in mice [2,5].

IGF-1, IGF-2 and insulin constitute a family of factors that regulate normal development and cellular function following initial binding to their dimeric cell surface receptor tyrosine kinases (IGF-1R, -2R and IR) [6]. IGF-1 and insulin and their receptors (IR and IGF-1R) are structurally closely related, but their actions result in markedly different downstream changes in different cell and tissue types. Although Insulin and IGF-1 cross-react with their non-cognate receptors, each receptor binds its own ligand with a 100- to 1000-fold higher affinity thus triggering a signaling cascade that regulates cell differentiation, apoptosis and, proliferation [7]. IGF-1 acting through its cognate receptor does not stimulate lipogenesis or rescue the lethal phenotype in mice that lacks the insulin receptor (IR) [8]. The insulin (IR) and IGF-1 receptors (IGF-1R) being structurally related, target several common intracellular substrates. However, each hormone also elicits specific effects through differential phosphorylation of their common substrates. For instance, differential phosphorylation of *FKHR*, a forkhead transcription factor occurs in response to signaling from insulin or IGF-1 receptor. In IR-deficient hepatocytes, one (Thr24) of the three phosphorylation sites in FKHR was not phosphorylated, though they express IGF-1R, resulting in distinctly different outcomes [9]. In addition, IGF-1 action is regulated via its interaction with multiple binding proteins [7,10].

A comparative microarray study investigating the effects of IGF-1 and insulin (employing ~2222 probe sets) has shown that 30 genes were specifically responsive to IGF-1 and 9 genes to insulin [11]. In mouse NIH-3T3 fibroblasts IGF-1 induced mitogenesis and/or differentiation whereas genes induced by insulin did not fall into any particular category [12]. Exposure of C2 myoblasts to a mutated IGF-1 derivative (for 24 hours) resulted in the differential regulation of about 90 genes [13]. Further the authors report identifying 28 muscle-specific as well as 33 un-annotated transcripts that are differentially expressed between IGF-1 and PDGF treatment of IGF-2-deficient murine C2 myoblasts.

In the present study we employed an Affymetrix mouse array platform (comprising ~22, 600 probe sets) to investigate the acute affects of exogenously added IGF-1 on global gene expression profiles in murine C2C12 myoblasts by exposing these for 1, 2 and 4 hours. The proportion of genes significantly affected by IGF-1 in this study is low (<1% of the whole genome) and belong to a mixed array of gene ontologies. Apart from the early induction or repression of transcription factors with IGF-1 treatment, a surprising finding was the coordinate induction of most genes of two related pathways, namely the fatty acid and cholesterol biosynthetic pathways. We therefore sought to determine whether this coordinate up-regulation of fatty acid and cholesterol biosynthetic genes in myoblasts, normally ascribed to as an insulin response, was modulated by the hierarchical lipogenic sterol regulatory element binding proteins (SREBPs). The SREBPs are not significantly transcriptionally induced with IGF-1 treatment. However, there is elegant evidence to suggest that these transcriptional factors are regulated by a complex post-transcriptional mechanism [14] and have been shown more recently by Brown and collaborators [15] to operate via Akt to induce ER-to-Golgi transport of the SREBP cleavage-activating protein (SCAP) and thereby stimulate SREBP processing.

## Results

### Global changes in gene expression

Murine C2C12 myoblast offers a robust model to study the acute effects of IGF-1 as expression patterns obtained here are indicative of the early changes leading to skeletal myotubule differentiation. We therefore investigated these global gene expression profiles following exposure of myoblasts to IGF-1 for 1, 2 and 4 hours. Correlation of data from the two independent experiments indicates reproducible expression signals for each partner time point at zero hour (data not shown). A similar correlation was also obtained for the other experimental time points. We tentatively selected a two-fold (or greater/lesser) criteria (relative to control values) to be a conservative threshold for measuring alterations in gene expression.

**Figure 1:**
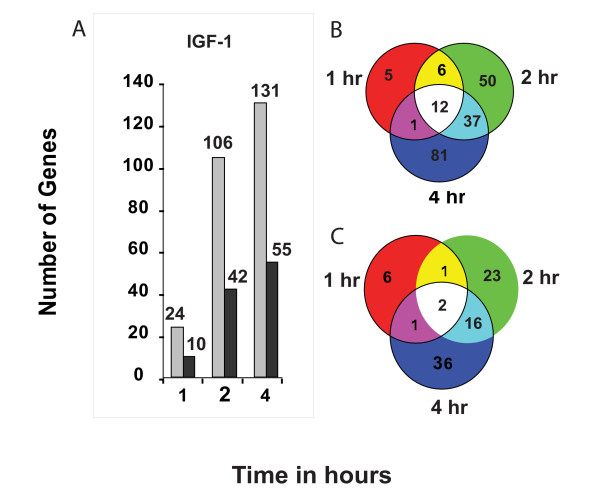
**Global changes in gene expression following exposure of murine myoblasts to IGF-1**. The number of genes that are either up- or down-regulated following acute exposure of C2C12 myoblasts to IGF-1 is shown (Fig. 1A). More genes are up- than down-regulated by IGF-1 at all the time points studied. Several, early induced genes are transcription factors and few of these are consistently expressed at all times. More genes are induced or repressed at the later times indicating secondary affects on the expression of a larger number of target genes, following the early surge in transcription factor expression. Figures 1 B-C show the temporal pattern of up- and down-regulated genes following exposure of murine myoblasts to IGF-1. Data from Venn diagrams show that several early expressed genes are transcription factors. Only 12 and 2 genes are up- (Fig. 1B) or down-(Fig. 1C) regulated, respectively, at all time points of acute IGF-1 treatment. At least 37 genes are induced at 2 hrs and remain so at 4 hrs. In contrast the expression of only 16 genes is repressed at 2 hrs and this remains so at 4 hrs. A tabulated list of genes for each of these groups is given elsewhere (see Tables 1 and 2 and also Additional files 1 and 2). Genes considered here and in other sections represent a 2-fold or greater change with respect to the zero time point.

Pair-wise comparisons of microarray data, generated from two independent experiments revealed that about twice the number of genes are differentially up-regulated by two-fold or greater levels as compared to genes whose expression is down regulated (Fig. 1A) at the 1, 2 and 4 hours of IGF-1 treatment and the number of genes showing increased expression increases through hour 4. The proportion of genes significantly affected by IGF-1 (2-fold or greater change) from the whole genome is low (<1%) demonstrating the specificity of IGF-1 action on a subset of genes. Though a number of the genes show some change (ie >5%) with IGF-1 treatment, they are not altered by the two-fold or greater criteria used here. On the other hand, the expression of about 400 genes was wholly unaffected (exhibiting < 5% change) with the treatment and these signal levels were indistinguishable from control samples at all time points. These unaffected genes belong to a mixed array of gene ontologies.

The temporal expression patterns of up- and down-regulated genes is shown in Venn distributions (Figs 1B–C) and the gene list for each overlapping and unique group is tabulated (see Tables 1 and 2 and Additonal files 1 and 2). The expression of 12 genes is both persistently and markedly up-regulated at all time points following IGF-1 treatment (Table 1). Genes such as Suppressor of cytokine signaling 3 (*Socs3*), DNA-damage inducible transcript 4 (*Ddit4*), Cation transport regulator-like 1(*Chac1*) and Hydroxysteroid (17 beta dehydrogenase 7 (*Hsd17b7*), were induced at all time points (Table 1 and Additional file 1). Only two genes, namely, Dual specificity phosphatase 1 (*Dusp1*) and Inhibitor of DNA binding 3 (*Idb3*), were consistently down-regulated at all times (Table 2 and Additional file 2).

**Table 1:** The temporal pattern of genes up-regulated with IGF-1 treatment

**Affy. Id**.	**Symbol**	**Genes up-regulated at 1 hr only**	**Fold Change**
1452519_a_at	*Zfp36*	Zinc finger protein 36	2.2
1450295_s_at	*Pvr*	Poliovirus receptor (pvr)	2.2
1427186_a_at	*Mef2a*	Myocyte enhancer factor 2A	2.2
1418102_at	*Hes1*	Hairy and enhancer of split 1	2.2
1416442_at	*Ier2*	Immediate early response 2	2.2
		**Genes up-regulated at 1 & 2 hrs**	
1427683_at	*Egr2*	Early growth response 2	2.6
1427174_at	*Phlda3*	Pleckstrin homology-like dom. family A, member 1	2.4
1419816_s_at	*Zfp36l2*	Zinc finger protein 36, C3H type-like 2	2.4
1418835_at	*Phlda1*	Pleckstrin homology-like dom. family A, member 1	2.4
1437626_at	*Zfp36l2*	Zinc finger protein 36, C3H type-like 2	2.2
1436026_at	*Zfp703*	zinc finger protein 703	2.2
		**Genes up-regulated at 1 & 4 hrs**	
1421077_at	*Sertad3*	SERTA domain containing 3	2.5
		**Genes up-regulated at 1, 2 & 4 hrs**	
1455899_x_at	*Socs3*	Suppressor of cytokine signalling 3	5.0
1451382_at	*Chac*	Cation transport regulator-like 1	5.0
1428306_at	*Ddit4*	DNA-damage-inducible transcript 4	4.0
1417871_at	*Hsd17b7*	Hydroxysteroid (17-beta) dehydrogenase 7	3.6
1424022_at	*Osgin1*	Oxidative stress induced growth inhibitor 1	3.3
1418025_at	*Bhlhb2*	Basic helix-loop-helix domain containing, class B2	3.3
1416029_at	*Klf10*	Kruppel-like factor 10	3.2
1424709_at	*Sc5d*	Sterol-C5-desaturase	3.1
1456212_x_at	*Socs3*	Suppressor of cytokine signaling 3	2.9
1448742_at	*Snai1*	Snail homolog 1	2.9
1448170_at	*Siah2*	Seven in absentia 2	2.7
1434204_x_at	*Shmt2*	Serine hydroxymethyl transferase 2 (mito)	2.2
		**Genes up-regulated at 2 hrs only**	
1421228_at	*Ccl7*	Chemokine (C-C motif) ligand 7	4.5
1422213_s_at	*Foxh1*	Forkhead box H1	4.5
1449227_at	*Ch25h*	Cholesterol 25-hydroxylase	4.0
1438097_at	*Rab20*	RAB20, member RAS oncogene family	3.3
1426706_s_at	*Xylb*	Xylulokinase homolog (H. influenzae)	3.3
1421215_a_at	*Slmap*	Sarcolemma associated protein	3.1
1428888_at	*Tmem33*	Transmembrane protein 33 clone	3.1
1448125_at	*Rit2*	Ras-like without CAAX 2	3.0
1452402_at	*Uchl3*	Ubiquitin carboxyl-terminal esterase L3	3.0
1452412_at	*Hoxc8*	Homeo box C8	2.9
1426958_at	*Rps9*	Ribosomal protein S9	2.9
1425624_at	*Epm2aip1*	EPM2A (laforin) interacting protein 1	2.8
1427583_at	*Rik*	RIKEN cDNA 4921505C17 gene	2.8
1441023_at	*Eif2s2*	Eukaryotic translation initiation factor 2 beta	2.7
1426065_a_at	*Trib3*	Tribbles homolog 3 (Drosophila)	2.6
425362_at	*Hrb1*	HIV-1 Rev binding protein-like	2.6
1424950_at	*Sox9*	SRY-box containing gene 9	2.6
1455904_at	*Gas5*	Growth arrest specific 5	2.5
1456094_at	*Usp36*	Ubiquitin specific peptidase 36	2.5
1449414_at	*Zfp53*	Zinc finger protein 53	2.5
1453806_at	*Ndufb2*	NADH dehydrogenase (ubiquinone) 1 beta subcomplex, 2	2.5
1417597_at	*Cd28*	CD28 antigen	2.5
1422851_at	*Hmga2*	High mobility group AT-hook 2	2.4
1450780_s_at	*Hmga2*	High mobility group AT-hook 2	2.4
1445116_at	*Usp25*	Ubiquitin-specific processing protease	2.4
1425500_x_at	*Coro2a*	Coronin, actin binding protein 2A	2.4
1449578_at	*Supt16h*	Suppressor of Ty 16 homolog	2.4
1449110_at	*Rhob*	Ras homolog gene family, member B	2.3
1425281_a_at	*Tsc22d3*	TSC22 domain family 3	2.3
1437658_a_at	*Snord22*	small nucleolar RNA, C/D box 22	2.3
1420961_a_at	*Ivns1abp*	Influenza virus NS1A binding protein	2.3
1421512_at	*Cep250*	Centrosomal protein 250	2.3
1453497_a_at	*Piga*	Phosphatidylinositol glycan, class A	2.3
1416693_at	*Foxc2*	Forkhead box C2	2.2
1450781_at	*Hmga2*	High mobility group AT-hook 2	2.2
1438527_at	*Rpl3*	Ribosomal protein L3	2.2
1448171_at	*Siah2*	Seven in absentia 2	2.2
1424607_a_at	*BC003993*	K0208G08-3 NIA Mouse clone	2.2
1420380_at	*Ccl2*	Chemokine (C-C motif) ligand 2	2.2
1417395_at	*Klf4*	Kruppel-like factor 4	2.2
1431030_a_at	*Rnf14*	Ring finger protein 14	2.2
1448183_a_at	*Hif1a*	Hypoxia inducible factor 1, alpha subunit	2.1
1419157_at	*Sox4*	SRY-box containing gene 4	2.1
1418158_at	*Trp63*	Transformation related protein 63	2.1
1421000_at	*Cnot4*	CCR4-NOT transcription complex, subunit 4	2.1
1452161_at	*Tiparp*	TCDD-inducible poly(ADP-ribose) polymerase	2.0
1425279_at	*Pdik1l*	PDLIM1 interacting kinase 1 like	2.0
1453840_at	*Pabpc1*	Poly A binding protein, cytoplasmic 1	2.0
1416123_at	*Ccnd2*	Cyclin D2	2.0
1417924_at	*Pak3*	P21-activated kinase 3	2.0

The temporal pattern of genes up-regulated in mouse myoblasts following IGF-1 treatment is tabulated based on their Venn distribution. Shown are genes up-regulated at: 1 hr only; 1 & 2 hrs; at 1 & 4 hrs; 1, 2 & 4 hrs and 2 hrs only (Please see Additional file 1 for genes up-regulated at 2 & 4 hrs and 4 hrs only).

**Table 2:** The temporal pattern of genes down-regulated with IGF-1 treatment

**Affy. Id**.	**Symbol**	**Genes down-regulated at 1 hr only**	**Fold Change**
1420019_at	*Tspan8*	Tetraspanin 8	0.41
1438317_a_at	*Endog*	Endonuclease G	0.44
1427298_at	*Dnm3os*	Dynamin 3, opposite strand	0.46
1456078_x_at	*Tubb2c*	Tubulin, beta 2c	0.46
1427543_s_at	*Ube1y1*	Ubiquitin-activating enzyme E1, Chr X	0.47
1438403_s_at	*Ramp2*	Receptor (calcitonin) activity modifying protein 2	0.48
		**Genes down-regulated at 1 & 2 hrs**	
1415996_at	*Txnip*	Thioredoxin interacting protein	0.47
		**Genes down-regulated at 1 & 4 hrs**	
1442744_at	*Rbm39*	RNA binding motif protein 39	0.45
		**Genes down-regulated at 1, 2 & 4 hrs**	
1448830_at	*Dusp1*	Dual specificity phosphatase 1	0.35
1416630_at	*Ib3*	Inhibitor of DNA binding 3	0.49
		**Genes (23) down-regulated at 2 hrs only**	
1422474_at	*Pde4b*	Phosphodiesterase 4B, cAMP specific	0.29
1435872_at	*Pim1*	Proviral integration site 1	0.32
1416488_at	*Ccng2*	Cyclin G2	0.35
1422473_at	*Pde4b*	Phosphodiesterase 4B, cAMP specific	0.36
1427005_at	*Plk2*	Polo-like kinase 2	0.37
1456569_x_at	*Gsn*	Gelsolin	0.39
1433668_at	*Pnrc1*	Proline-rich nuclear receptor coactivator 1	0.41
1419080_at	*Gdnf*	Glial cell line derived neurotrophic factor	0.42
1416286_at	*Rgs4*	Regulator of G-protein signaling 4	0.44
1460009_at	*Ier5*	Immediate early response 5	0.45
1422195_s_at	*Tbx15*	T-box 15	0.46
1448364_at	*Ccng2*	Cyclin G2	0.47
1416619_at	Rik	RIKEN 4632428N05 gene	0.47
1450741_at	*Stau1*	Staufen (RNA binding protein) homolog 1	0.47
1427479_at	*BB287469*	Eukaryotic translation initiation factor1A, predicted	0.48
1434940_x_at	*Rgs19*	Regulator of G-protein signaling 19	0.48
1437101_at	*Lats2*	LATS2B, alternatively spliced	0.49
1452604_at	*Stard13*	Serologically defined colon cancer antigen 13	0.49
1456528_x_at	*Ncl*	Nucleolin	0.49
1439441_x_at	*Lats1*	Large tumor suppressor 2	0.49
1427130_x_at	Rik	RIKEN 1700021K02 gene	0.49
1451731_at	*Abc1*	ATP-binding cassette, sub-family A, member 3	0.49
1453355_at	*Wnk2*	WNK lysine deficient protein kinase 2	0.50

The temporal pattern of genes down-regulated in mouse myoblasts following IGF-1 treatment is tabulated based on their Venn distribution. Shown are genes down-regulated at: 1 hr only; 1 & 2 hrs; at 1 & 4 hrs; 1, 2 & 4 hrs and 2 hrs only (Please see Additional file 2 for genes down-regulated at 2 & 4 hrs and 4 hrs only).

The early induction events target a battery of transcription factors which include, Early growth response 1 & 2 (*Egr-1/-2*), Snail homolog 1(*Snai1*), Basic h-l-h domain containing class B2 (*Bhlhb2*), Zinc finger protein-36 (*Zfp36*), -97-like (*Zfp97l*), -119 (*Zfp119*), Kruppel-like factor 10 (*Klf10*), and Immediate early response-2 (*Ier2*) genes, that are all up-regulated following 1 hour of treatment. Of these early responding transcription factors some, such as the Early growth response-1 and -2 (*Egr-1 *and -*2*), Zn finger proteins-36 (*Zfp36*) and -119 (*Zfp119*), Immediate early response (*Ier2*) and DNA-damage inducible transcript 4 (*Ddit4*) gene are transiently expressed (See Table 1) exhibiting reduced expression after the initial surge. Other early-induced genes, for example snail homolog 1 (*Snai1*), Seven in absentia 2 (*Siah2*), Kruppel-like factor 10 (*Klf10*), Basic helix-loop-helix domain containing factor (*Bhlhb2*), remain so for the rest of the treatment.

### Coordinate expression of fatty acid and cholesterol biosynthetic pathway genes

**Figure 2:**
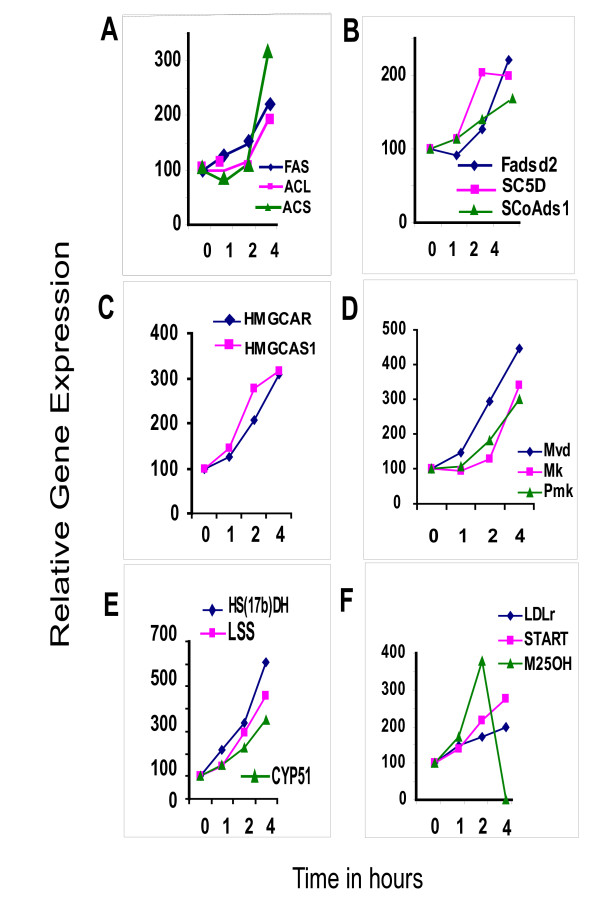
**Expression profiles of fatty acid and cholesterol biosynthetic pathway genes following exposure of murine myoblasts to IGF-1**. The expression values (derived from microarray studies) are plotted as relative change over untreated control (100) values for Fatty acid synthase (Fas), ATP citrate lyase (Acly), Acyl CoA synthetase (Acs) (Fig. 2 A; Fatty acid desaturase (Fads), sterol C5 desaturase (SC5d), and Stearoyl CoA desaturase 1 (Scd1) (Fig. 2B). Data shows a two-fold or greater induction with IGF-1 treatment for all these genes (Fig. 2 A-B). Relative gene expression profiles are also shown for the genes involved in cholesterol biosynthesis (Fig 2C–F), namely HMG CoA synthase 1 (Hmgcs1) and HMG CoA reductase (Hmgcr) (Fig. 2C); Mevalonate (diphospho) decarboxylase (Mvd), Mevalonate kinase (Mk) and Phosphomevalonate kinase (Pmk) (Fig. 2D). Profiles are also shown for Cyp51, Hydroxysteroid dehydrogenase 17 beta (Hsd17b7), Lanosterol synthase (Lss) (Fig. 2E) and for the Low density Lipoprotein receptor (Ldlr), START domain 4 (Startd4) and cholesterol 25-hydroxylase (M25oh) genes (Fig. 2F). The genes involved in fatty acid and cholesterol biosynthesis are coordinately induced by IGF-1, though subtle variations exist in the time and extent of induction. In general, the induction is about 3–4 fold.

At 4 hours of treatment IGF-1 induced marked changes in two major pathways; those for 5 genes of the fatty acid and 19 genes of the cholesterol biosynthetic pathways. The induced fatty acid genes include ATP citrate lyase (*Acly*), Acetyl CoA synthase (*Acs*), Long chain Elongase (*Lce*), Fatty acid synthase (*Fas*), Stearoyl-CoA desaturase 1 (*Scd1*)(Figs. 2A, B and Table 3). The key fatty acid regulatory gene, Acetyl CoA carboxylase alpha (*Acc1*) did not respond to IGF-1 treatment, though its signal levels were scored as 'Present' under Affymetrix' signal selection categories of 'Present (P), Absent (A), or Marginally (M) present'.

**Table 3:** Fatty Acid biosynthetic pathway genes up-regulated with IGF-1 treatment

**Affy. Id**.	**Symbol**	**Genes up-regulated at 4 hrs**	**Fold Change**
1425326_at	*Acyl*	ATP citrate lyase *	2.7
1418911_s_at	*Acas1*	Acyl CoA synthetase *	1.9
1427595_at	*Acat1*	Acetyl-CoA carboxylase	N/C
1423828_at	*Fas*	Fatty acid synthase	2.3
1451457_at	*Sc5dl*	Delta-5 desaturase	2.0
1415824_at	*Scd1*	Steroyl CoA desaturase	1.8
1417404_at	*Evol6*	Elongation of Long chain FA-6	2.3

The genes involved in fatty acid biosynthesis that are induced by about two-fold or greater levels at 4 hours are listed. Only a selective number of fatty acid genes are induced by IGF-1. Acetyl-CoA carboxylase a key and rate-limiting enzyme in the pathway shows no detectable transcriptionally induction. Genes common to the fatty acid and cholesterol biosynthetic pathways (see Table 4) are indicated (*) and genes showing 'No Change' in expression levels are also listed (N/C).

The cholesterol biosynthetic pathway comprises multiple enzymatic steps leading to cholesterol biosynthesis and these genes are coordinately up-regulated by IGF-1 (Fig. 2 and Table 4). The key cholesterol biosynthetic enzymes, namely HMG CoA reductase (*Hmgcr*) (~3.1-fold) (Fig. 2C) and 7 dehydrocholesterol reductase (*7Dhcr*) (~3.2-fold) are up-regulated to similar extents. With the exception of the Hydroxysteroid dehydrogenase (*Hsd-17b*) (Fig. 2E), which is induced to over 6-fold levels, the other inducible cholesterol pathway genes are stimulated to 3- to 4-fold levels relative to untreated samples. In general, initial changes in expression levels for most fatty acid and cholesterol pathway genes is perceptible at 2 hours and is more pronounced at 4 hours of treatment (Fig. 2 and Table 4). The expression of sterol-C5 desaturase (*Sc5d*) and Hsdh7 (Fig 2B and Fig 2E) genes, however, is significantly induced at the earliest time point and remains induced for the entire period of treatment.

**Table 4:** Cholesterol biosynthetic pathway genes up-regulated with IGF-1 treatment

**Affy. Id**.	**Symbol**	**Genes up-regulated at 4 hrs**	**Fold Change**
1425326_at	*Acly*	ATP Citrate Lyase *	2.7
1422478_a_at	*Acas1*	Acetyl CoA Synthetase *	3.3
1451271_a_at	*Acat*	Acetyl CoA Acetyltransferase	N/C
1423797_at	*Acas*	Acetyl CoA Acety Synthetase	3.1
1433443_at	*Hmgcs1*	HMG CoA Synthase 1	3.1
1427229_at	*Hmgcr*	HMGCoA Reductase	3.1
1430619_a_at	*Mvk*	Mevalonate Kinase	3.9
1427893_a_at	*Pmek*	Phosphomevalonate Kinase	3.0
1417303_at	*Mvd*	Mevalonate (diphospho) Decarboxylase	4.5
1451122_at	*Idi1*	Isopentenyl diphosphate delta isomerase	2.9
1423418_at	*Fdps*	Farnesyl diphosphate Synthetase	1.8
1415993_at	*Sqe*	Squalene epoxidase	2.1
1426913_at	*Lss*	Lanosterol Synthase	4.6
1422533_at	*Cyp51*	Lanosterol 14a demethylase (CYP51)	3.5
1423078_a_at	*Sc4mo*	Sterol C4 Methyl Oxidase	3.5
1416222_at	*Nsdhl*	NAD(P)H Steroid Dehydrogenase-like	2.4
1417871_at	*Hsd17b7*	17b Hydroxysteroid Dehydrogenase 7	6.1
1424709_at	*Sc5d*	Sterol C5 Desaturase	3.0
1448619_at	*7Dhcr*	7 dehydrocholesterol Reductase	3.2
		**Uptake, breakdown & transport genes**	
1421821_at	*Ldlr*	LDL Receptor (Uptake)	2.0
1449227_at	*M25oh*	Cholesterol 25 hydroxylase (breakdown)	4.0
1429240_at	*Startd4*	Star-related Lipid transfer domain containing 4 (Transport)	2.8
		**Regulatory genes**	
1426690_a_at	*Srebp1a*	Sterol Regulatory Element Binding Protein-1	1.1
1426744_at	*Srebp2*	Sterol Regulatory Element Binding Protein-2	1.4
1433520_at	*Scap*	SREBP Cleaveage Activator Protein	N/C
1448240_at	*S1p*	Site 1 protease	N/C
1417980_a_at	*Insig2*	Insulin Signal 2 (insulin induced)	1.7

The genes involved in cholesterol biosynthesis that are induced by about two-fold or greater levels at 4 hours are listed. All genes involved in cholesterol biosynthesis as well as those involved in cholesterol uptake (LDL receptor), break down (cholesterol 25 hydroxylase (M25oh)) and transport (Star-related Lipid transfer domain containing 4(Startd4)), are also up-regulated with IGF-1 treatment. Of the genes involved in the regulation of cholesterol biosynthesis only Srebp2 and Insig2 are feebly up-regulated, whereas Scap and S1p genes are unaffected. There is no available chip data on S2p. Genes common to the fatty acid and cholesterol biosynthetic pathways are indicated (*) and genes showing 'No Change' in expression levels are also listed (N/C).

Interestingly, the LDL receptor (*Ldlr*) (~2-fold), Star-related Lipid transfer domain containing 4 (*Startd4*) (2.8-fold) and cholesterol 25 hydroxylase (*M25oh*) (~4-fold) genes that are involved in cholesterol uptake, transport, and breakdown, respectively, are also up-regulated in the same time frame (Fig 2F and Table 4).

**Figure 3:**
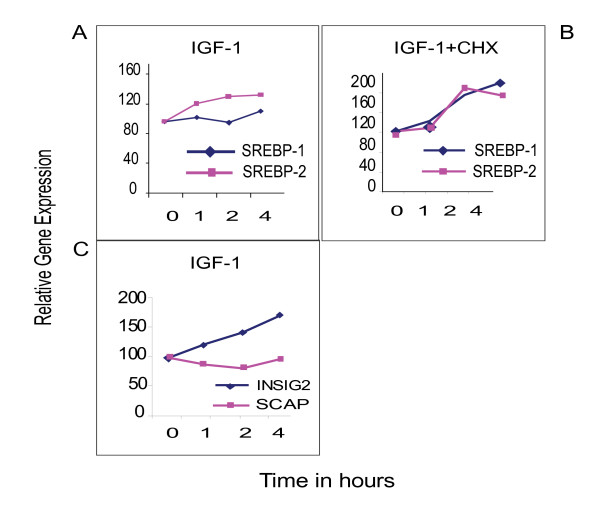
**Genes involved in the regulation of fatty acid and cholesterol biosynthetic pathways**. Microarray data show no significant change in SREBP-1 and -2 gene expression with IGF-1 treatment (Fig. 3A). However, when cycloheximide was added in conjunction with IGF-1 (IGF-1+CHX) to block nascent protein synthesis, both genes were activated to low and comparable extents, suggesting a similar pattern of regulation via 'derepression' (Fig. 3B). This level of 'derepression' was not seen with CHX treatment alone (data not shown). Insig2, Scap, S1p and S2p, are genes associated with SREBP-mediated lipogenesis, of these only Insig2 expression is moderately (1.6-fold) up-regulated (Fig 3C).

**Figure 4:**
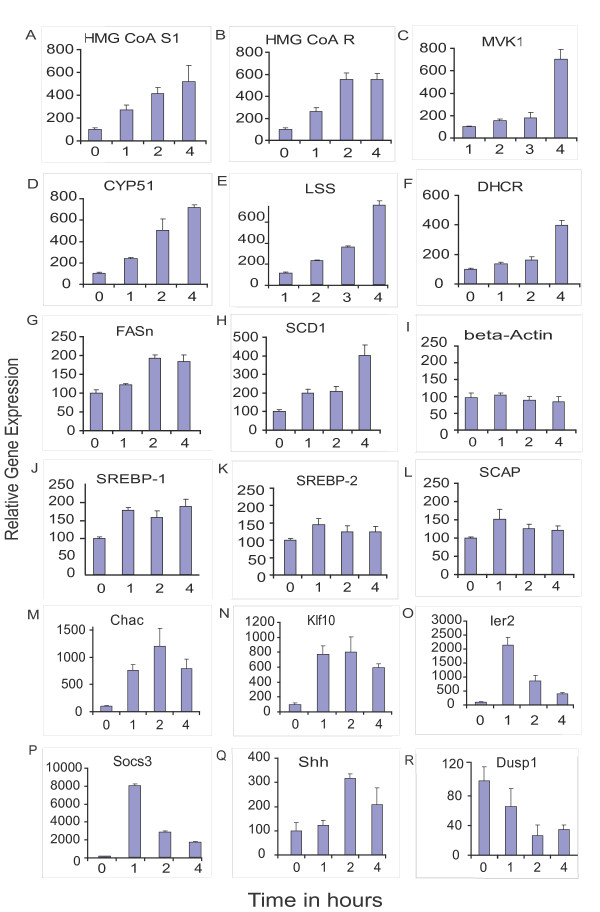
**Qantitative-PCR based assay for selective fatty acid, cholesterol biosynthetic pathway and regulatory genes following IGF-1 treatment**. The relative abundance of mRNA as compared to untreated control samples was assayed by q-PCR and plotted on a histogram for 6 genes involved in the cholesterol biosynthetic pathway, namely for HMG CoA synthase 1 (Hmgcs1), HMG CoA reductase (Hmgcr), (Mevalonate kinase (Mk/Mvk1), Cytochrome P450 51 (Cyp51), Lanosterol synthetase (Lss), and Dehydrocholestrol reductase (7Dhcr) (Fig. 4 A–F); 2 genes involved in fatty acid biosynthesis, namely Fatty Acid synthase (Fas) and stearoyl-Coenzyme A desaturase 1 (Scd1)(Fig. 4G and 4H); and 3 regulatory genes involved in fatty acid and cholesterol biosynthesis, namely SREBP-1 and -2, and Sterol Cleavage Activator Protein (SCAP) (Fig. 4J, 4K and 4L). The beta-Actin mRNA was assayed here as a control (Fig. 4I). In addition, 6 other genes unrelated to the fatty acid or cholesterol pathways were assayed; namely Chac1, Klf10, Ier2, Socs3, Shh and Dusp1 (Fig. 4 M-R, respectively) and the data show that the expression profile for all of these genes is similar to patterns obtained from our microarray experiments. The pattern obtained is in general agreement with the Affymetrix GeneChip data for these genes, with the exception of SREBP-1 where no change was detected with the microarray study. The extent of induction obtained with q-PCR, however, is greater than the microarray hybridization signals, probably due to the greater dynamic range observed with PCR amplification.

Of the many transcriptional factors implicated in the regulation of fatty acid and cholesterol biosynthesis it was of interest to examine the two principal factors SREBP-1 and -2 (Fig. 3A, B). No measurable change was observed for the SREBP-1 gene and no significant change (1.4-fold increase) was observed for the SREBP-2 gene with IGF-1 treatment. The q-PCR data for SREBP-1 is at variance with the microarray studies (Fig. 4J). SREBP-1 and -2 are not significantly induced by IGF-1 treatment alone. However, both SREBP-1 and -2 are significantly induced when IGF-1 is treated in the presence of the cycloheximide (Fig. 3B), a potent inhibitor of protein synthesis. The expression of other genes involved in the post-transcriptional regulation of SREBPs, such as SREBP Cleavage Activator Protein (*Scap*) (Fig. 3C and 4L) and, Site 1 protease (*S1p*) were not induced, with the exception of *Insig2 *which again was moderately (1.6-fold) stimulated by IGF-1 (Fig. 3C)(No chip data was available for the Site 2 protease (*S2p*)). Furthermore, none of the other known transcription factors/cofactors implicated in fatty acid and cholesterol biosynthesis, namely *Ap1, AP2, Sp1, Sp3, Lxr*, *C/EBPbeta*, *NF-Y*, and *Red25 *were induced by IGF-1 treatment in this study.

Microarray data was validated using q-PCR on a selection of 6 cholesterol biosynthetic pathway genes, namely HMG CoA synthase 1 (*Hmgcs1*), HMG CoA reductase (*Hmgcr*), (Mevalonate kinase (*Mk*), Cytochrome P450 51 (*Cyp51*), Lanosterol synthetase (*Lss*), 7 Dehydrocholestrol reductase (*7Dhcr*) (Fig. 4A–F, respectively). A comparison of the microarray and q-PCR data for each of these genes shows that the profiles are similar. However, the extent of induction seen with q-PCR is more profound. For instance, microarray data for HMG CoA synthase and HMG CoA reductase (see Fig. 2C) indicates a 3-fold change whereas q-PCR values show a 4- to 5-fold induction at 4 hours (Figs. 4A, B). Likewise the relative expression values obtained with the other cholesterol biosynthetic pathway genes is greater though the profiles are quite similar. This similarity is further exemplified when a comparison of data from the two analytical methods is made for *Mk, Lss *and *Cyp51 *genes. The *Mk (Mvk1) *gene is markedly induced at 4 hours with both methods but remains largely uninduced at the earlier time points (see Figs. 2D and 4I). However, induction levels obtained by q-PCR for the *Mvk1 *gene at 4 hours (Fig. 4C) are about 2-fold greater than that obtained with microarray studies. Further, microarray data for both LSS and *Cyp51 *genes show a more or less linear induction with IGF-1 that is initiated at 1 hour (Fig. 2E) and is unlike the profile obtained for the *Mvk1 *gene. Again our qPCR data shows a similar pattern of induction for both genes though the change is about 2-fold greater at 4 hours (Figs. 4D and 4E).

The fold change profiles for 2 representative genes of the fatty acid biosynthesis pathway, namely Fatty Acid synthase (*Fas*) and stearoyl-Coenzyme A desaturase 1 (*Scd1*) (Figs. 4G and 4H, respectively) are similar with both methods. The *Scd1 *gene, and not *Fas*, shows a greater level of stimulation with the q-PCR assay. Three regulatory genes involved in fatty acid and cholesterol biosynthesis, namely SREBP-1 & -2, and Sterol Cleavage Activator Protein (*Scap*) were examined (Figs. 4J, K, L respectively). The pattern obtained is in general agreement with the Affymetrix GeneChip data for these genes, with the exception of SREBP-1 where no change was detected with the microarray study (Fig. 3A) as against a measurable change detected with the q-PCR assay (Fig. 4J).

The beta-Actin gene included as an independent control here (Fig. 4I) showed no change with IGF-1 treatment. Further, we selected five genes unrelated to the fatty acid or cholesterol biosynthetic pathways, namely Chac1, Klf10, Ier2, Socs3 and Shh (Fig 4M, N, O, P and 4Q, respectively) that show distinctive patterns of early induction based on our microarray data. In addition, we selected Dusp1, a dual-specificity phosphatase gene (Fig. 4R), which is one of the two genes that are consistently down-regulated over the IGF-1 treatment regime, to reflect a gene that was repressed by IGF-1 treatment. The q-PCR profiles for all these six genes are shown (Fig. 4M to 4R.) and closely resemble the characteristic expression patterns observed for each of these genes from our microarray studies with the exception that Shh shows a slightly delayed induction. Overall, the extent of change obtained with q-PCR was invariably greater than that noticed with the microarray studies.

## Discussion

### Global changes in gene expression

Insulin characteristically influences the up- and down- regulation of more than 150 genes in various tissues and induces lipogenesis in muscle cells [16]. Several target genes induced by insulin are not affected by IGF-1 treatment at any time point in this study. For instance, *Glut2 *(glucose transport); Glucokinase, aldolase A, phosphoglycerate kinase and GAPDH (glycolysis); Glucose-6 phosphate dehydrogenase (pentose phosphate); leptin and Apolipoprotein A1 (lipid transport); calmodulin (Calcium signaling) and Plasminogen activator inhibitor-1 (*Pai1*) (fibrinolysis) genes [16] that are characteristically up-regulated by insulin are not altered by IGF-1 treatment. In contrast, we have identified a subset of genes typically activated by insulin, namely *VEGF, Glut1, IGF-1, IGFBP-3*, fatty acid synthesis genes, ATP-citrate lyase, Fatty acid synthase, Stearoyl CoA desaturase 1, cholesterol synthesis and uptake genes, HMG CoA reductase, LDL receptor and *Egr-1 *transcription factor gene, that are also activated by IGF-1 in this study. Moreover two genes, GAPDH and *Pai1 *[16] that are induced by insulin were markedly down-regulated (2.6- and 1.8-fold, respectively) by IGF-1 in our study.

Conversely, several liver-specific genes, known to be down-regulated by insulin including Phospho-enolpyruvate carboxykinase (*PEPCK*), the rate limiting enzyme in gluconeogenesis; 3-Hydroxyl-3-methylglutaryl-CoA synthase-2 (*Hmgcs2*) involved in ketogenesis; IGFBP-1 carrier protein; Pyruvate dehydrogenase kinase-4 (*Pdk4*) involved in inhibition of lipogenesis, and two cytochromes P450, *Cyp7 *and *Cyp8B1*, are all unaffected by IGF-1 treatment. In contrast to the down-regulation of 5-aminolevulinate synthase-1 (*ALAS1*), the key and rate-limiting enzyme in heme biosynthesis by insulin, IGF-1 induces its expression (1.9-fold). Despite the structural similarities between the insulin and IGF-1 ligands and their receptors, the downstream pathways affected by these two hormones in myoblasts are different in important respects and follow divergent action streams. The divergent effects of insulin and IGF-1 were reported earlier in a microarray study on mouse NIH-3T3 fibroblasts where intracellular signals for these two peptide hormones are different [12].

Alternative measures such as quantitative PCR are widely used to validate gene expression data obtained with microarrays. We used q-PCR methods to assay selective genes involved in the fatty acid and cholesterol biosynthetic pathways as well as genes not directly related to these two pathways that were either induced or repressed (Fig. 4) and found the results to be in general agreement with the Affymetrix microarray chip data in that the direction and pattern of change were closely similar for most genes, though the fold change seen with q-PCR was generally greater. These findings are also in agreement with earlier reports that q-PCR validations are directional confirmation only and large discrepancies in the amount of change are observed [17].

One of the early responses to IGF-1 treatment was that the genes for a significant number of transcription factors were markedly induced. As indicated earlier several of these factors such as Early growth response-1 & -2, Zinc finger protein-36, -97-like, -119 (*Zfp36, Zfp97l, Zfp119*), and Immediate early response-2 genes (See Table 1), were transiently expressed. The expression of other transcription factors such as Snail homolog 1 (*Snai1*), Seven in absentia 2 (*Siah2*), Kruppel-like factor 10 (*Klf10*), Basic helix-loop-helix domain containing factor (*Bhlhb2*) is sustained for the period of treatment. A large number of genes are either up- or down-regulated and belong to a mixed array of gene ontologies. However, it was interesting that some genes belonging to the fatty acid biosynthetic pathway and most genes of the cholesterol biosynthetic pathway were coordinately up-regulated at 4 hours following exposure of murine C2C12 myoblasts to IGF-1(Tables 3 and 4).

Only a few of the 30 genes reported by Dupont and coworkers [11] from their 90 min study on IGF-1 treated NIH3T3 cells are up-regulated in our study on C2C12 myoblasts; namely, the Early growth response 1 and a Splicing factor, arginine/serine 3, gene. C2 cells treated for 24 hours with an IGF-1 analogue R3-IGF-1 were shown to up-regulate about 90 genes, of which 28 were muscle-specific [13]. The large number of muscle-specific genes induced by this prolonged exposure to IGF-1 (or its analogue) is consistent with its role in myoblast differentiation. The lack of a similarity between the above two reports and the present study with mouse C2C12 myoblasts is likely due to the differences in cell types, induction times and IGF-1 employed in the respective studies, and therefore it is not surprising that there are few common patterns.

### Regulation of Fatty Acid and Cholesterol genes

Insulin has been implicated in triggering lipogenesis and most evidence suggests that this stimulation is brought about via the induction of the SREBPs and other partner proteins associated with their transcription as well as post-transcriptional regulation. There is no transcriptional evidence to suggest that this coordinate up-regulation of lipogenesis in myoblasts, normally considered an insulin response, is modulated by the hierarchical SREBPs. For instance the SREBP-1 transcription factor, implicated in the induction of fatty acid biosynthesis [14], was not transcriptionally activated by IGF-1 in our microarray study (Figs. 3A). Although, SREBP-1 is reported to self-induce its own transcription by a 'feed-forward loop' mechanism [18] we failed to see any significant transcriptional enhancement in myoblasts. Further, SREBP-2 is modestly (1.4-fold) affected by IGF-1 in our microarray (Fig. 3A) (and q-PCR, Fig. 4K) study. The related SREBP cleavage activating protein (SCAP) (Fig. 3C and Fig. 4L) and the membrane bound Site 1 and 2 peptidases (S1p and S2p) are not transcriptionally induced. However, INSIG2, which triggers SREBP cleavage, is moderately (1.6-fold) induced (Fig. 3C). It should be pointed out that SREBP-1a and -1c mRNAs differ only in their 5' coding regions (SREBP-1a mRNA encodes 28 additional N-terminal amino acids whereas SREBP-1c lacks this region but has 4 unique amino acids. The mRNA sequences downstream from this region are identical in both isoforms). The Affymetrix mouse chip and our q-PCR analysis target the common 3' region of SREBP-1a and SREBP-1c and hence relate to both isoforms (-1a & -1c). However, it is reported that SREBP-1a is a potent activator of gene expression as compared to the relatively weak inducer activity associated with SREBP-1c [19].

Several transcription factors are implicated in insulin-mediated regulation of fatty acids and cholesterol biosynthesis, including AP1, AP2, SREBP-1a, -1c and -2, Sp1, Sp3, LXR, C/EBPbeta, NF-Y, and Red25 [16]. None of the aforementioned factors record a change in their relative gene expression signal levels with 4 hours of IGF-1 treatment. An alternative explanation is that the post-transcriptional regulation of SREBPs, as outlined in several earlier studies [14], could account for the coordinate induction of fatty acid and cholesterol biosynthetic genes following IGF-1 treatment.

Tjian and coworkers [20] have reported that the CREB-binding protein (CBP) and p300 (a CBP-related protein, CrP) are transcriptional coactivators that interact with SREBP promoters. Coexpression with p300 dramatically increases the expression of both SREBP-1a and -2 [21]. Our data indicate that neither of the co-activators, CBP or p300 is up-regulated. It is reported that cholesterol biosynthesis depends almost entirely on SREBPs whereas fatty acid synthesis is only partially dependent on these factors [18]. However, the lack of induction of these coactivators (CBP or p300) with IGF-1 treatment may account for the poor transcriptional induction of SREBPs and further support the proposal that post-transcriptional pathways operate to induce fatty acid and cholesterol biosynthesis in myoblasts.

SREBPs are the master regulators of lipid homeostasis and SREBP-1 and SREBP-2 are known to preferentially up-regulate genes involved in fatty acid or cholesterol biosynthesis, respectively [22]. Brown and coworkers (see [15]) in a series of interesting experiments in CHO cells involving IGF-1, used a PI3K inhibitor to inactivate Akt or expressed a dominant-negative form of Akt and have reported that IGF-1 induced fatty acid and cholesterol biosynthesis by a process mediated via the PI3K/Akt pathway. More interestingly, IGF-1 induces the transport of SCAP, the SREBP cleavage-activating protein that escorts SREBP from the endoplasmic reticulum to the Golgi by a process which can be blocked with LY294002, which inhibits PI3K and can, thereby, affect Akt activity. The cleavage of SREPBs to release the active transcriptional factor operates through the stimulation of the PI3K/Akt pathway by IGF-1, which in turn induces the transport of SCAP and SREBP to the Golgi and eventually results in the processing of SREBP. Processed SREBPs are localized to the nucleus to ultimately trans-activate fatty acid and cholesterol genes. It is therefore plausible that the IGF-1-mediated induction of fatty acid and cholesterol biosynthesis pathways elaborately delineated by Brown and coworkers [15] is likely to operate by a wholly post-transcriptional process and occurs as an early stimulatory event in IGF-1 treated myoblasts that are devoid of any significant transcriptional expression of SREBPs. It is also possible that other post-transcriptional mechanisms operate similar to the insulin-dependent phosphorylation of SREBP-1c that was recently shown to promote its transcriptional activity [23].

## Conclusion

In summary, the present studies suggest that the acute action of IGF-1 in murine myoblast, besides inducing and repressing an array of genes of diverse ontologies as presented here, also brings about the coordinate induction of several fatty acid and cholesterol biosynthetic pathway genes, probably through the trans-activation of the hierarchical SREBP transcription factors through a previously elucidated subtle post-transcriptional mechanism that occurs via Akt, initiating the transport of SCAP to the Golgi and leads to increased proteolytic activation of SREBPs. Another important avenue to explore is whether other factors cooperatively influence lipogenesis in myoblasts together with the SREBPs, following induction by IGF-1.

## Methods

The following reagents were commercially obtained: Mouse skeletal myoblast cell line, C2C12 (ATCC, CRL 1772); Delbeco's Modified Eagle Media (DMEM), fetal calf serum (FCS) and PBS from Gibco; recombinant human IGF-1 from Chemicon (Temecula, CA); cycloheximide (CHX) from Calbiochem (San Diego, CA); oligonucleotide pairs for q-PCR were chemically synthesized by ValueGene (San Diego, CA); SuperScript First Strand Synthesis System from InVitrogen (Carlsbad, CA, USA); Quantitech Syber Green PCR kit from Qiagen (Valencia, CA). RNA was isolated using the RNAeasy and QIAshredder kits from Qiagen and prepared for hybridization using the Message Amp II aRNA kit from Ambion (Austin, Tx); bio-11-CTP and Bio-16-UTP were purchased from Enzo Life Sciences (Farmingdale, NY). The GeneChip Mouse Expression Array 430A from Affymetrix (Santa Clara, CA) was used in these studies. All other reagents were of molecular biology grade.

### Cell culture

Mouse C2C12 myoblasts were grown to confluency in 100 mm plates and maintained in DMEM containing 10% FCS. Prior (10 min) to treatment, media were aspirated from culture dishes and cells were washed twice with 1× PBS to remove residual serum. Cells were incubated in serum-deprived DMEM media (2 ml) at 37°C with 5% C0_2 _for 1, 2 and 4 hours, to which one of the following conditions was added: a) 1× PBS; b) IGF-1; c) IGF-1+CHX or d) CHX alone. A zero time point sample was also included. The final concentrations of recombinant IGF-1 and cycloheximide were 100 ng/ml and 100 μg/ml, respectively. The dose of IGF-1 selected was close to physiological concentration (range 75–125 ng/ml). Each set of treatment conditions was prepared in batches prior to addition to triplicate culture plates and the entire experimental set was independently repeated. Samples of culture media were screened prior to RNA isolation for mycoplasma (UCSD Microplasma Core Facility) and were found to be free from contamination.

### Isolation and labeling of RNA

At each time point, batches of cell culture plates were washed in cold PBS (1×), aspirated and rapidly frozen with liquid nitrogen and stored at -80°C. Cells were harvested by scraping with a rubber policeman and total RNA was extracted using the Qiagen RNeasy kit according to manufacturer's instructions. The quality and quantity of total RNA samples pooled (from triplicate plates) were examined using an Agilent 2100 Bioanalyzer. Single and double stranded cDNA were prepared from the total RNA using Ambion's Message Amp II kit. Briefly, 4 μg of mRNA was used to generate first-strand cDNA by using a T7-linked oligo(dT) primer. After second-strand synthesis, T7 polymerase directed *in vitro *transcription was performed in the presence of biotin-labeled UTP and CTP (Enzo Life Sciences) to generate biotin-incorporated cRNA using Ambion's Message Amp II cRNA amplification system. A complete description of these procedures are available at the Ambion website . The quality and purity of duplicate cRNA samples were again assessed with the Agilent 2100 Bioanalyzer. The cRNA (samples pooled from triplicate plates each derived from a duplicate set of experiments) were used in a duplicate set of arrays.

The UCSD GeneChip Core Facility performed dye labeling, fragmentation, hybridization, washing and subsequent scanning of the arrays according to the procedures recommended by the manufacturer . All experiments were performed using the Affymetrix Mouse Expression 430A oligonucleotide arrays, using protocols as described on the manufacturer's website . The Affymetrix Mouse 430A chip contains primarily probe sets against well annotated full-length genes. The target cRNA generated was processed as per the recommendations of the manufacturer . Controls were spiked to 10 μg of fragmented cRNA samples and these were hybridized overnight using the Affymetrix Hybridization Oven 640. Arrays were then washed and stained with streptavidin-phycoerythrin using the Affymetrix GeneChip Fluidics Station 450 and finally scanned using an Affymetrix GeneChip^® ^Scanner 3000-7G. A complete description of these procedures is available at . After scanning, array images were assessed to confirm scanner alignment and the absence of significant bubbles or scratches on the chip surface. The 3'/5' ratios for GAPDH and beta-actin were confirmed to be within acceptable limits (< 3.0). The BioB spike controls were found to be present on all chips and, the BioC, BioD and CreX controls were present in increasing intensities. Scaling factors for all arrays were within acceptable limits as were background, Q values and mean intensities.

### Data Analysis

Scanned array images were converted to intensity values for each probe and chip using the Affymetrix MAS 5.0 software and arrays that met the acceptable Affymetrix criteria were used for further analysis. All microarray data were scaled to a standard target intensity of 500 using Affymetrix's MAS 5.0 software. The raw data were transferred to the GeneSpring (Agilent Technologies/Silicon Genetics) or VAMPIRE (variance-modeled posterior inference with regional exponentials) microarray suite [24] for data analyses. The one-color (Affymetrix) data from duplicate hybridization experiments were normalized on a per-chip and per-gene basis using the GeneSpring protocol, filtered for 'Present (P) only' genes, then for fold-change (2-fold or greater) over zero time point values and finally the relative expression levels plotted for the different time points for selected genes. Differentially expressed genes reported here, using the GeneSpring Computer Software, are based on average signal intensities indicating up- (i.e. 2-fold or greater) or down-regulation (0.5 or less) following IGF-1 treatment, as compared to untreated samples at the different time points. Raw data below a signal threshold (of 100) were generally filtered out as background noise unless the profiles indicated reproducible and markedly higher signal levels at some other time point of treatment. All expression data are based on averaged normalized relative fold change or averaged raw signal intensity values. Data presented here conforms to the proposed MIAME criteria [25] and checklist . Comparisons using the Vampire Software and the accompanying Goby gene ontology database, are based on differential expression of duplicate set of genes between treated and control samples, that are statistically significant (p = < 0.05). The fold change (2-fold or greater) values presented here are corrected to one and two decimal places for the up- and down-regulated genes, respectively.

### Quantitative-PCR

Validation of microarray expression data was carried out using quantitative PCR for selective genes (see Fig. 4). For this study, cDNA was generated from the pooled replicate samples of total RNA using SuperScript First Strand Synthesis enzyme (InVitrogen) for the initial reverse transcription reaction. Custom-synthesized (Valuegene, San Diego, CA) gene-specific oligonucleotide pairs were used. Four replicate q-PCR reactions were set up for each time point, comprising cDNA samples, SyberGreen Master Mix (Qiagen), RNAse-free water and the appropriate primer pair in a final volume of 20 ml. Suitable volumes of pooled mixes were prepared and aliquots were used in the replicate tubes to minimize experimental error. Reactions were carried out in a MJ Research Instrument (Opticon 2, Bio-Rad, Carlsbad, CA) and included the following steps; denaturation for 15 seconds at 94°C; annealing for 30 seconds at 55°C; extension for 30 seconds at 72°C for 35 cycles.

## Abbreviations

CHX: cycloheximide; IGF-1: insulin-like growth factor 1; IR: insulin receptor; IGF1R: IGF-1 receptor; INSIG1: insulin stimulated gene 1; SRE: sterol regulatory element; SREBP: SRE binding protein; SCAP: sterol cleavage activator protein; S1p & S2p: Site 1 & 2 protease, respectively. Abbreviation for genes (gene symbols) are tabulated alongside the full names in the appropriate tables.

## Authors' contributions

The contributions of CRB comprise the design and conducting of research, analysis of data and writing the manuscript. TF was involved in the analysis of data and reviewed drafts of the manuscript.

## Supplementary Material

Additional file 1 **The temporal pattern of up-regulated genes in mouse myoblasts following IGF-1 treatment.** Shown are genes up-regulated at 2 & 4 hrs and, 4 hrs only. Click here for file

Additional file 2 **The temporal pattern of down-regulated genes in mouse myoblasts following IGF-1 treatment. **Shown are genes down-regulated at 2 & 4 hrs and, 4 hrs only. Click here for file
