# Supplementary material for: Insulin-like growth factor-1 coordinately induces the expression of fatty acid and cholesterol biosynthetic genes in murine C2C12 myoblasts
Source: BMC Genomics. 2008 Nov 11;9:535. doi: 10.1186/1471-2164-9-535 (PMC2628395; doi:10.1186/1471-2164-9-535)
Supplement: Additional file 1 — The temporal pattern of up-regulated genes in mouse myoblasts following IGF-1 treatment. Shown are genes up-regulated at 2 & 4 hrs and, 4 hrs only. [file 1471-2164-9-535-S1.doc]

Additional files

Additional file 1

File format: DOC

| Title: The temporal pattern of up-regulated genes in mouse myoblasts following IGF-1 treatment | | | |
| --- | --- | --- | --- |
| Description: Shown are genes up-regulated at 2 & 4 hrs and, 4 hrs only. | | |  |
|  |  |  |  |
|  |  | **Genes up-regulated at 2 and 4 hrs** |  |
| 1426913_at | *Lss* | Lanosterol synthase | 4.7 |
| 1417303_at | *Mvd* | Mevalonate (diphospho) decarboxylase | 4.6 |
| 1448663_s_at | *Mvd* | Mevalonate (diphospho) decarboxylase | 4.5 |
| 1422533_at | *Cyp51* | Cytochrome P450, family 51 | 3.6 |
| 1423078_a_at | *Sc4mol* | Sterol-C4-methyl oxidase-like | 3.6 |
| 1448619_at | *7Dhcr* | 7-dehydrocholesterol reductase | 3.4 |
| 1420013_s_at | *Lss* | Lanosterol synthase | 3.3 |
| 1448865_at | *Hsd* | Hydroxysteroid (17-beta) dehydrogenase 7 | 3.3 |
| 1433446_at | *Hmgcs1* | 3-hydroxy-3-methylglutaryl-Coenzyme A synthase 1 | 3.2 |
| 1427229_at | *Hmgcr* | 3-hydroxy-3-methylglutaryl-Coenzyme A reductase | 3.2 |
| 1418937_at | *Dio2* | Deiodinase, iodothyronine, type II | 3.2 |
| 1416313_at | *Mllt11* | Myeloid/lymphoid or mixed-lineage leukemia (trithorax homolog) | 3.1 |
| 1418288_at | *Lpin1* | Lipin 1 | 3.1 |
| 1435357_at | *D4Wsu53e* | Chr 4, Wayne St Univ 53, expr | 3.0 |
| 1451776_s_at | *Hod* | Homeobox only domain | 3.0 |
| 1451122_at | *Idi1* | Isopentenyl-diphosphate delta isomerase | 3.0 |
| 1448700_at | *G0s2* | G0/G1 switch gene 2 | 2.9 |
| 1429239_a_at | *Stard4* | StAR-related lipid transfer (START) domain containing 4 | 2.8 |
| 1429240_at | *Stard4* | StAR-related lipid transfer (START) domain containing 4 | 2.8 |
| 1432032_a_at | *Artn* | Artemin | 2.6 |
| 1434877_at | *Nptx1* | Neuronal pentraxin 1 | 2.5 |
| 1450646_at | *Cyp51* | Cytochrome P450, family 51 | 2.5 |
| 1419666_x_at | *Nupr1* | Nuclear protein 1 | 2.4 |
| 1433576_at | *Mat2a* | Methionine adenosyltransferase II, alpha | 2.4 |
| 1422612_at | *Hk2* | Hexokinase 2 | 2.3 |
| 1420493_a_at | *Pcyt2* | Phosphate cytidylyltransferase 2, ethanolamine | 2.3 |
| 1456702_x_at | *Mat2a* | Methionine adenosyltransferase II, alpha | 2.2 |
| 1426259_at | *Pk3* | Pantothenate kinase 3 | 2.2 |
| 1417516_at | *Ddit3* | DNA-damage inducible transcript 3 | 2.2 |
| 1432181_s_at | *Ecgf1* | Endothelial cell growth factor 1 (platelet-derived) | 2.1 |
| 1415784_at | *Vps35* | Vacuolar protein sorting 35 | 2.1 |
| 1420342_at | *Gdap10* | Ganglioside-induced differentiation-associated-protein 10 | 2.1 |
| 1438630_x_at | *Mat2a* | Methionine adenosyltransferase II, alpha | 2.1 |
| 1433613_at | *Pank3* | Pantothenate kinase 3 | 2.0 |
| 1456380_x_at | *Cnn3* | Calponin 3 | 2.0 |
| 1438386_x_at | *Mat2a* | Methionine adenosyltransferase II, alpha | 2.0 |
| 1451457_at | *Sc5d* | Sterol-C5-desaturase | 2.0 |
|  |  | **Genes up-regulated at 4 hrs only** |  |
| 1416481_s_at | *Higd1a* | HIG1 domain family, member 1A | 3.7 |
| 1415996_at | *Txnip* | Thioredoxin interacting protein | 3.6 |
| 1418052_at | *Mvk* | Mevalonate kinase | 3.5 |
| 1450188_s_at | *Lipg* | Lipase, endothelial | 3.5 |
| 1422478_a_at | *Acss2* | Acyl-CoA synthetase short-chain fly. Memb. 2 | 3.3 |
| 1456081_a_at | *Aacs* | Acetoacetyl-CoA synthetase | 3.2 |
| 1423797_at | *Aacs* | Acetoacetyl-CoA synthetase | 3.2 |
| 1427893_a_at | *Pmvk* | Phosphomevalonate kinase | 3.1 |
| 1426616_at | *Tlcd1* | TLC domain containing 1 | 3.1 |
| 1454161_s_at | *Rik* | RIKEN 0610007P14 gene (Steroid biosynthesis) | 3.0 |
| 1451461_a_at | *Aldoc* | Aldolase 3, C isoform | 2.9 |
| 1418829_a_at | *Eno2* | Enolase 2, gamma neuronal | 2.8 |
| 1417860_a_at | *Spon2* | Spondin 2, extracellular matrix protein | 2.8 |
| 1425326_at | *Acly* | ATP citrate lyase | 2.8 |
| 1423804_a_at | *Idi1* | Isopentenyl-diphosphate delta isomerase | 2.8 |
| 1421830_at | *Ak3l1* | Adenylate kinase 3 alpha-like 1 | 2.7 |
| 1426516_a_at | *Lpin1* | Lipin 1 | 2.7 |
| 1437722_x_at | *Pcbp3* | Poly(rC) binding protein 3 (Pcbp3), mRNA | 2.6 |
| 1418129_at | *Dhcr24* | 24-dehydrocholesterol reductase | 2.6 |
| 1419391_at | *Myog* | Myogenin | 2.6 |
| 1448130_at | *Fdft1* | Farnesyl diphosphate farnesyl transferase 1 | 2.6 |
| 1435639_at | *Rik* | RIKEN cDNA 2610528A11 gene | 2.5 |
| 1433428_x_at | *Tgm2* | Transglutaminase 2, C polypeptide | 2.5 |
| 1435630_s_at | *Acat2* | Acetyl-Coenzyme A acetyltransferase 2 | 2.5 |
| 1420772_a_at | *Tsc22d3* | TSC22 domain family 3 | 2.5 |
| 1418648_at | *Egln3* | EGL nine homolog 3 | 2.5 |
| 1450383_at | *Ldlr* | Low density lipoprotein receptor | 2.4 |
| 1416432_at | *Pfkfb3* | 6-Phosphofructo-2-kinase/fructose-2,6-biphosphatase 3 | 2.4 |
| 1416222_at | *Nsdhl* | NAD(P) dependent steroid dehydrogenase-like | 2.4 |
| 1422479_at | *Acss2* | Acyl-CoA synthetase short-chain fly. memb. 2 | 2.4 |
| 1448752_at | *Car2* | Carbonic anhydrase 2 | 2.4 |
| 1417404_at | *Elovl6* | ELOVL fam. memb. 6, elongation of long chain fatty acids | 2.4 |
| 1456731_x_at | *Polr3k* | Polymerase (RNA) III (DNA directed) polypeptide K | 2.4 |
| 1417890_at | *Pdxp* | Pyridoxal (pyridoxine, vitamin B6) phosphatase | 2.4 |
| 1419248_at | *Rgs2* | Regulator of G-protein signaling 2 | 2.3 |
| 1416151_at | *SRp20* | Splicing factor, arginine/serine-rich 3 | 2.3 |
| 1449893_a_at | *Lrig1* | Leucine-rich repeats and immunoglobulin-like domains 1 | 2.3 |
| 1417403_at | *Elovl6* | ELOVL fam. Memb. 6, elongation of long chain fatty acids | 2.3 |
| 1433640_at | *Fubp1* | Far upstream element (FUSE) binding protein 1 | 2.3 |
| 1455494_at | *Col1a1* | Procollagen, type I, alpha 1 | 2.3 |
| 1417122_at | *Vav3* | Vav 3 oncogene | 2.3 |
| 1418760_at | *Rdh11* | Retinol dehydrogenase 11 | 2.3 |
| 1424140_at | *Gale* | Galactose-4-epimerase, UDP | 2.3 |
| 1423828_at | *Fas* | Fatty acid synthase | 2.3 |
| 1450417_a_at | *Rps20* | Ribosomal protein S20 | 2.3 |
| 1436944_x_at | *Rik* | Phosphatidylserine decarboxylase, pseudogene | 2.2 |
| 1424984_at | *Rik* | RIKEN cDNA 2700078E11 | 2.2 |
| 1424243_at | *Rwdd4a* | RWD domain containing 4A | 2.2 |
| 1421679_a_at | *Cdkn1a* | Cyclin-dependent kinase inhibitor 1A (P21) | 2.2 |
| 1419050_at | *Rik* | RIKEN cDNA 1110002H13 gene | 2.2 |
| 1416035_at | *HIF1a* | Hypoxia inducible factor 1, alpha subunit | 2.2 |
| 1451666_at | *Acly* | ATP citrate lyase | 2.2 |
| 1451895_a_at | *Dhcr24* | 24-dehydrocholesterol reductase | 2.2 |
| 1422692_at | *Sub1* | SUB1 homolog | 2.2 |
| 1418649_at | *Egln3* | EGL nine homolog 3 | 2.2 |
| 1415993_at | *Sqle* | Squalene epoxidase | 2.2 |
| 1422977_at | *Gp1bb* | Glycoprotein Ib, beta polypeptide | 2.2 |
| 1438322_x_at | *Fdft1* | Farnesyl diphosphate farnesyl transferase 1 | 2.2 |
| 1426988_at | *Klhdc5* | Kelch domain containing 5 | 2.2 |
| 1438366_x_at | *Clcn3* | Chloride channel 3 transcript variant a | 2.2 |
| 1419703_at | *Col5a3* | Procollagen, type V, alpha 3 | 2.2 |
| 1422155_at | *Hist2h3c2* | Histone 2, H3c2 | 2.2 |
| 1421469_a_at | *Stat5a* | Signal transducer & activator of transcription 5A | 2.2 |
| 1440221_at | *AA408650* | Transcribed locus | 2.1 |
| 1448371_at | *Mylpf* | Myosin light chain, phosphorylatable, fast skeletal muscle | 2.1 |
| 1428662_a_at | *Hod* | Homeobox only domain | 2.1 |
| 1428083_at | *Rik* | RIKEN cDNA 2310043N10 gene | 2.1 |
| 1423924_s_at | *Tspan14* | Tetraspanin 14 | 2.1 |
| 1419551_s_at | *Stk39* | Ser/Thr kinase 39, STE20/SPS1 homolog | 2.1 |
| 1449142_a_at | *Yipf5* | Yip1 domain family, member 5 | 2.1 |
| 1423071_x_at | *Rik* | RIKEN clone:6720475J19 | 2.1 |
| 1438154_x_at | *Rik* | RIKEN cDNA 2610002J02 gene | 2.1 |
| 1428091_at | *Kl7* | Kelch-like 7 | 2.1 |
| 1420760_s_at | *Ndrg1* | N-myc downstream regulated-like | 2.1 |
| 1418949_at | *Gdf15* | Growth differentiation factor 15 | 2.1 |
| 1425195_a_at | *Acat3* | Acetyl-Coenzyme A acetyltransferase 3 | 2.1 |
| 1421821_at | *Ldlr* | Low density lipoprotein receptor | 2.0 |
| 1428392_at | *Rassf2* | Ras association (RalGDS/AF-6) domain fly.2 | 2.0 |
| 1425280_at | *Leng1* | Leukocyte receptor cluster (LRC) member 1 | 2.0 |
| 1415964_at | *Scd1* | Stearoyl-Coenzyme A desaturase 1 | 2.0 |
| 1424633_at | *Camk1g* | Calcium/calmodulin-dependent protein kinase Iγ | 2.0 |
