# Supplementary material for: Insulin-like growth factor-1 coordinately induces the expression of fatty acid and cholesterol biosynthetic genes in murine C2C12 myoblasts
Source: BMC Genomics. 2008 Nov 11;9:535. doi: 10.1186/1471-2164-9-535 (PMC2628395; doi:10.1186/1471-2164-9-535)
Supplement: Additional file 2 — The temporal pattern of down-regulated genes in mouse myoblasts following IGF-1 treatment. Shown are genes down-regulated at 2 & 4 hrs and, 4 hrs only. [file 1471-2164-9-535-S2.doc]

| Additional file 2 |  |  |  |
| --- | --- | --- | --- |
| File format: DOC |  |  |  |
| Title: The temporal pattern of down-regulated genes in mouse myoblasts following IGF-1 treatment | | | |
| Description: Shown are genes down-regulated at 2 & 4 hrs and, 4 hrs only. | | |  |
|  |  |  |  |
|  |  | **Genes (16) down-regulated at 2 & 4 hrs** |  |
| 1416953_at | *Ctgf* | Connective tissue growth factor | 0.15 |
| 1416039_x_at | *Cyr61* | Cysteine rich protein 61 | 0.16 |
| 1438133_a_at | *Cyr61* | Cysteine rich protein 61 | 0.17 |
| 1435458_at | *Pim1* | Proviral integration site 1 | 0.18 |
| 1452387_a_at | *Amotl2* | Angiomotin like 2 | 0.21 |
| 1420992_at | *Ankrd1* | Ankyrin repeat domain 1 | 0.23 |
| 1417522_at | *Fbxo32* | F-box only protein 32 | 0.26 |
| 1423006_at | *Pim1* | Proviral integration site 1 | 0.26 |
| 1420991_at | *Ankrd1* | Ankyrin repeat domain 1 | 0.27 |
| 1448747_at | *Fbxo32* | F-box only protein 32 | 0.30 |
| 1423100_at | *Fos* | FBJ osteosarcoma oncogene | 0.32 |
| 1421267_a_at | *Cited2* | Cbp/p300-interacting transactivator (Glu/Asp-rich C-term. dom2) | 0.35 |
| 1416041_at | *Sgk* | Serum/glucocorticoid regulated kinase | 0.39 |
| 1438387_x_at | *Top3b* | Topoisomerase (DNA) III beta | 0.42 |
| 1452207_at | *Cited2* | Cbp/p300-interacting transactivator (Glu/Asp-rich C-term. dom, 2) | 0.42 |
| 1419728_at | *Cxcl5* | Chemokine (C-X-C motif) ligand 5 | 0.50 |
|  |  | **Genes (36) down-regulated at 4 hr only** |  |
| 1451612_at | *Mt1* | Metallothionein 1 | 0.26 |
| 1420973_at | *Arid5b* | AT rich interactive domain 5B (Mrf1 like) | 0.29 |
| 1417065_at | *Egr1* | Early growth response 1 | 0.29 |
| 1438674_a_at | *Sfrs8* | Splicing factor, arginine/serine-rich 8 | 0.35 |
| 1422537_a_at | *Id2* | Inhibitor of DNA binding 2 | 0.36 |
| 1442745_x_at | Rbm39 | RNA binding motif protein 39 | 0.36 |
| 1436871_at | Sfrs7 | Splicing factor, arginine/serine-rich 7 | 0.37 |
| 1426951_at | *Crim1* | Cysteine rich transmembrane BMP regulator 1 | 0.37 |
| 1421365_at | *Fst* | Follistatin | 0.39 |
| 1418293_at | *Ifit2* | Interferon-induced protein with tetratricopeptide repeats 2 | 0.39 |
| 1435176_a_at | *Id2* | Inhibitor of DNA binding 2 | 0.39 |
| 1450377_at | *LOC640441* | Thrombospondin 1, similar to | 0.40 |
| 1438138_a_at | *Pex6* | Peroxisomal biogenesis factor 6 (Pex6) | 0.41 |
| 1422168_a_at | *Bdnf* | Brain derived neurotrophic factor | 0.42 |
| 1420275_at | *Rik* | RIKEN clone 9030017G24 | 0.44 |
| 1422912_at | *Bmp4* | Bone morphogenetic protein 4 | 0.44 |
| 1427031_s_at | *Ccdc52* | Coiled-coil domain containing 52 | 0.45 |
| 1446147_at | *Rbm39* | RNA binding motif protein 39 | 0.45 |
| 1419184_a_at | *Fhl2* | Four and a half LIM domains 2 | 0.45 |
| 1452449_at | *Hmbox1* | RIKEN hypothetical protein | 0.46 |
| 1435133_at | *Ugcg* | UDP-glucose ceramide glucosyltransferase | 0.46 |
| 1421446_at | *Prkcc* | Protein kinase C, gamma | 0.46 |
| 1432478_a_at | *Ibrdc3* | IBR domain containing 3 | 0.46 |
| 1426624_a_at | *Ypel3* | Yippee-like 3 (Drosophila) | 0.47 |
| 1452180_at | *Phf17* | PHD finger protein 17 | 0.47 |
| 1436032_at | Rik | RIKEN clone:I830083F22 | 0.47 |
| 1453748_a_at | *Kif23* | Kinesin family member 23 | 0.47 |
| 1423690_s_at | *Gpsm1* | G-protein signalling modulator 1 | 0.48 |
| 1422053_at | *Inhba* | Inhibin beta-A | 0.48 |
| 1426870_at | *Fbxo33* | F-box only protein 33 | 0.48 |
| 1448390_a_at | *Dhrs3* | Dehydrogenase/reductase member 3 | 0.48 |
| 1424942_a_at | *Myc* | Myelocytomatosis oncogene | 0.49 |
| 1422243_at | *Fgf7* | Fibroblast growth factor 7 | 0.49 |
| 1448890_at | *Klf2* | Kruppel-like factor 2 | 0.49 |
| 1449292_at | *Rb1cc1* | RB1-inducible coiled-coil 1 | 0.49 |
| 1436922_at | *Ppil5* | Peptidylprolyl isomerase (cyclophilin) like 5 | 0.50 |
